# Comparative study on *in vitro* fertilization, cleavage and blastocyst development of post-warmed vitrified cattle oocytes in different media

**DOI:** 10.3389/fvets.2025.1722882

**Published:** 2025-11-18

**Authors:** Maleke Dimpho Sebopela, Ntuthuko Raphael Mkhize, Mamonene Angelinah Thema, Masindi Lottus Mphaphathi

**Affiliations:** 1 Germplasm Conservation and Reproductive Biotechnologies, Agricultural Research Council, Pretoria, South Africa; 2 Animal Science Discipline, School of Agriculture and Science, College of Agriculture, Engineering and Science, University of KwaZulu-Natal, Durban, South Africa; 3 Department of Agriculture and Animal Health, University of South Africa College of Agriculture and Environmental Sciences, Johannesburg, South Africa

**Keywords:** cryopreservation, immature oocytes, pronucleus, cleavage, cattle

## Abstract

**Introduction:**

Improving the efficiency of in vitro embryo production could be possible by optimizing the media used for oocyte cryopreservation, fertilization, and embryo culture. This study evaluated the fertilization success and developmental competence of post-warmed vitrified cattle oocytes cultured in three distinct in vitro protocols.

**Methods:**

A total of 270 immature oocytes were randomly divided into three experimental groups (n = 90 per group), each exposed to different vitrification, warming and fertilization protocols. In Group 1; oocytes were vitrified and warmed using commercial (ART Lab Solutions, Australia) medium, Group 2; oocytes were vitrified and warmed using TCM 199-based solution (prepared medium) and Group 3; oocytes were vitrified and warmed using Bioscience^™^ medium (IVF Bioscience, BO-VitriCool^™^ and BO-Vitri Warm^™^, United Kingdom). Post-warming, oocytes were matured in vitro per group: Group 1—Vitromat-Protect^™^, Group 2—TCM199, and Group 3—BO IVM^™^. Matured oocytes were then subjected to in vitro fertilization (IVF) using VitroFert^™^ (Group 1), prepared BO-IVF (Group 2) and BO-IVF^™^ (Group 3). Following 18 h post-IVF, presumptive zygotes were washed and cultured in VitroCleave PLUS^™^ (Group 1), BO IVC (Group 2) and BO IVC^™^ (Group 3) media. Embryos were further cultured in BO IVC, BO IVC^™^, or VitroBlast (Group 1) protocol with medium changes as per respective protocols. These observations were repeated 6 times.

**Results:**

Among vitrified oocytes, BO IVF^™^ achieved the highest total (35.33 ± 3.61) and normal fertilization rates (42.66 ± 9.43), significantly higher than VitroFert^™^ and BO IVF. Non-vitrified oocytes showed no significant fertilization differences across media but consistently had higher cleavage rates (38.83–55.28%) than vitrified oocytes (26.50–30.00%). Embryos from non-vitrified oocytes cultured in BO IVC^™^ reached the highest morula (31.14 ± 6.17) and blastocyst (29.57 ± 6.97) development, whereas embryos from vitrified oocytes cultured in VitroCleave Plus^™^ yielded the highest morula and blastocyst rates (18.33 ± 15.7) compared with BO IVC^™^ (12.16 ± 14.14) and BO IVC (9.66 ± 15.18; *p* < 0.05).

**Conclusion:**

BO IVF^™^, and VitroCleave Plus^™^ medium demonstrated potential for enhancing post-warmed oocyte competence and fertilization, cleavage and blastocyst outcomes compared to other treatment groups. Although vitrification reduces fertilization and development, VitroCleave Plus^™^ appears to offer the optimal support for post-warmed oocytes to reach the blastocyst stage.

## 1 Introduction

*In vitro* production of embryos (IVEP) from cryopreserved oocytes is an important technique in assisted reproductive biotechnology, contributing substantially to animal breeding programs and advancing fundamental research in developmental physiology (1–3). A notable advantage of IVEP is the efficient use of semen, as a single straw can fertilize more than two hundred oocytes (4). Similar to how artificial insemination using frozen semen enhances the reproductive efficiency of bulls, oocyte cryopreservation can improve the reproductive potential of females with high genetic merit (2). Despite these advantages, oocytes are particularly difficult to cryopreserve due to their large size and low surface to volume ratio (5, 6). Vitrification, a cryopreservation method characterized by high concentrations of cryoprotectants (CPAs) and ultra-rapid cooling, prevents ice crystal formation and is therefore the recommended method for preserving large-volume cells such as oocytes (7). The efficiency of vitrification depends on factors such as the type and concentration of CPAs, the temperature of the vitrification solution, and the exposure duration before plunging into liquid nitrogen (8). Common CPAs include dimethylsulfoxide (DMSO), ethylene glycol (EG), and glycerol, used individually or in combination to protect cells and tissues from freezing damage. Unlike the thawing method, which uses a single rehydration step at room temperature, the warming process begins with an initial rehydration at 38.5 °C, followed by one or more rehydration steps at progressively lower CPA concentrations at room temperature (9). Most warming solutions are made with sucrose or trehalose in smaller amounts (10). Nevertheless, producing transferable bovine embryos from vitrified oocytes remains challenging to date (11–13). The drive to increase the efficiency of IVEP, particularly from cryopreserved oocytes, has motivated extensive applied research in embryo biology and culture. For IVEP, oocytes can be collected from slaughterhouse-derived ovaries or by follicular aspiration from live donor animals (14, 15). Cumulus–oocyte complexes (COCs), consisting of the oocyte surrounded by cumulus cells, are selected for further processing. The IVEP process involves four key steps: oocyte cryopreservation, *in vitro* maturation (IVM), fertilization (IVF), and culture (IVC) of embryos under controlled laboratory conditions (1, 4). Each stage requires carefully optimized cryopreservation protocols, culture media, and incubation conditions. Successful IVEP is influenced by three critical factors: the quality of oocytes, the effectiveness of cryopreservation, and the suitability of culture media to support proper development after IVM, IVF, and IVC (16). Despite the development of several cryopreservation, IVF, and IVC protocols for cattle IVEP, success rates remain inconsistent. Much of this variability arises from differences in laboratory protocols, particularly in the use of vitrification, fertilization, and culture media (17). Currently, the most commonly utilized fertilization media in cattle IVEP are BO-IVF^™^ (Biosciences) and TCM-based formulations. In the present study, we also evaluated Vitrofert^™^, an Australian-developed fertilization medium, for its potential to improve fertilization outcomes. To the best of our knowledge, this is the first application of Vitrofert^™^ in South African cattle IVEP protocols, particularly for post-warmed vitrified oocytes.

A major challenge in livestock breeding remains the preservation of oocyte viability and developmental competence following cryopreservation, as cryo-induced structural and functional damage can compromise fertilization and embryo development, thereby reducing production efficiency (18, 19). These outcomes are strongly influenced by the choice of cryopreservation protocol and embryo culture media, since variations in cooling/warming rates, cryoprotectant composition, and post-warm culture conditions can significantly affect oocyte survival, fertilization success, and subsequent embryonic development (20). Optimizing oocyte cryopreservation, fertilization, and embryo culture media could enhance IVEP efficiency (21). Therefore, the aim of this study was to evaluate the fertilization success and subsequent embryo developmental competence of post-warmed vitrified cattle oocytes across three distinct *in vitro* protocols, with specific focus on fertilization rates, cleavage progression, and blastocyst development.

## 2 Materials and methods

### 2.1 Chemicals, culture media, and culture conditions

All chemicals for *in vitro* cultures and analyses were purchased from Sigma-Aldrich (Milan, Italy), unless otherwise indicated.

### 2.2 Ovaries collection and cumulus oocytes complexes recovery

Heterogeneous beef cattle ovaries of unknown reproductive status were collected from a local abattoir. The ovaries were immediately transported to the Agricultural Research Council, Germplasm Conservation and Reproductive Biotechnologies (ARC, GCRB) laboratory in 0.9% saline water (SABAX pour saline Adcock Ingram, RSA) in a thermos flask at 37 °C. The ovaries temperature was checked using a thermometer (Pencil LASEC SA LAST12/20110 memmert, RSA). The ovaries were placed in a water bath (B. Owen Jones LTD, Macdonald Adams & Company, RSA) at 37 °C. The aspiration method for oocytes retrieval was carried out using 10 mL disposable syringes (U-Life-Medical, RSA) and an 18-gauge sterile hypodermic needle (U-Life-Medical, RSA). The needle was pushed inside the ovaries and sucked out the follicular fluid of visible follicles. The recovered follicular fluid was searched for the recovered oocytes under the stereo microscope (Olympus CX 23) (New York microscope Co, United States).

### 2.3 Vitrification and warming of cumulus oocytes complexes

Three vitrification and warming protocols were used: The vitrification procedure was performed at room temperature. The retrieved immature oocytes exhibiting multiple compact cumulus cell layers and homogeneous cytoplasm were selected as COCs for further processing. The oocytes were subsequently divided into non-vitrified and vitrified groups. The oocytes were subjected to cryopreservation using the conventional straw vitrification method and were exposed to different equilibration and vitrification solutions (VSs). A total of 270 oocytes were randomly divided into three experimental groups (*n* = 90 per group), each exposed to distinct vitrification and warming protocols. Group 1; oocytes were vitrified using ART Lab Solutions^™^ protocol. Initially, oocytes were held in handling medium (HM), consisting of base medium (BM) supplemented with 5 mg/mL bovine serum albumin (BSA), for 5–10 min. They were then exposed to VS 1 (containing BM with 5 mg/mL BSA, 7.5% EG, and 7.5% DMSO) for 3 min. This was followed by a 30 s exposure to VS 2, composed of 1 M sucrose with 16.5% EG and 16.5% DMSO. Following vitrification, oocytes were then loaded into French mini-straws 0.25 mL straws in the following order: A column of VS, an air bubble, a column of VS containing five to ten oocytes, an air bubble and a column of VS. The straws were pre-cooled by placing them horizontally on a styrofoam rack exposed 5 cm above the liquid nitrogen (LN_2_) vapour for 5 min. The oocyte’s frozen straws were loaded on the aluminium cryocane, then stored inside the LN_2_ tank (−196 °C) until warming. These observations were repeated 6 times per treatment. For warming, oocytes were sequentially transferred through four warming solutions: Warm 1 (800 μL HM + 400 μL sucrose medium [SM]) for less than 1 min, Warm 2 (800 μL HM + 400 μL SM) for 5 min, Warm 3 (800 μL HM + 200 μL SM) for another 5 min, and Warm 4 (800 μL HM) for 1 to 5 min before further processing.

The oocytes of Group 2 (*n* = 90) were exposed to TCM 199 (our prepared media) VS. The vitrification and warming of oocytes using the TCM199-based protocol was carried out as follows: Oocytes were first equilibrated in a base medium consisting of TCM199 supplemented with 10% fetal bovine serum (FBS) for 1 min. This was followed by rinsing in a buffered solution (BS) containing 7.5% FBS for 3 min. The oocytes were then transferred to a holding medium composed of BS supplemented with 20% FBS, 7.5% DMSO, and 7.5% EG for 3 min. For vitrification, oocytes were placed in a solution containing TCM199 with 20% FBS, 1.35% EG, 1.35% DMSO, and 0.5 M sucrose for 30 s. Following vitrification, oocytes were then loaded into French mini-straws 0.25 mL straws as described above. Warming was performed by immersing the oocytes in modified Dulbecco’s phosphate-buffered saline (mDPBS) supplemented with 20% FBS and 0.25 M sucrose for 30 s. Finally, oocytes were rehydrated in mDPBS containing 20% FBS and 0.15 M sucrose for 1 min before further proceeding to IVM and IVF.

The Group 3 oocyte vitrification and warming were performed using the BO-VitriCool^™^ and BO-VitriWarm^™^ kits (IVF Bioscience, United Kingdom), following the manufacturers protocol. Briefly, oocytes were first equilibrated at room temperature in BO-VitriCool^™^ 1, a serum-free medium containing EG and DMSO, for approximately 2 min. They were then transferred to BO-VitriCool^™^, also containing EG and DMSO, for an additional 2 min. This was followed by a final exposure to BO-VitriCool^™^ 3 for less than 1 min. Following vitrification, oocytes were then loaded into French mini-straws 0.25 mL straws as described above. These observations were repeated 6 times per treatment. For warming, oocytes were sequentially transferred through four warming solutions (BO-VitriWarm^™^) at 38.5 °C, each containing sucrose and albumin in a serum-free base. The oocytes were held in BO-VitriWarm^™^ 1 for 3 min, then in BO-VitriWarm^™^ 2 and 3 for 2 min each, and finally in BO-VitriWarm^™^ 4 for 1 min before proceeding to IVM and IVF.

### 2.4 Warming of the cryopreserved immature oocytes

Frozen oocyte straws were removed from the LN_2_ tank (−196 °C) and exposed to 10 s in the air, then plunged into the thawing container. The straws were exposed to warm (37 °C) water for 1 min during the thawing. The oocyte straws were cut at both ends and emptied into the thawing solutions to remove the intra-cellular cryoprotectant. A first sterile four-well dish was filled with medium and used as follows: oocytes were washed thrice in M199 + 10% FBS to remove the intracellular cryo-protectant and further processed to each warming medium as explained in section 2.3.

### 2.5 *In vitro* maturation of cattle oocytes

Post-warming vitrified and non-vitrified oocytes were subjected to IVM under the same media and culture conditions. The protocol used from IVM through to IVC was consistent for both groups and the number of oocytes allocated to each group was kept consistent to ensure comparability of results. The oocytes were washed six times before maturation: three times in mDPBS and three times in M199 + 10% FBS. The four-well dishes (Thermo Scientific Nunclon Delta surface Sigma-Aldrich, United States) were used for IVM. Each well contained 500 μL of maturation medium: (1) TCM199, containing medium 199 + 10% FBS supplemented with follicle stimulating hormone (FSH), luteinizing hormone, and estradiol hormone, sodium pyruvate, and antibiotics, (2) BO-IVM^™^, which is serum-free, supplemented with low glucose, gonadotrophic hormones, and gentamycin; (3) Vitromatprotect^™^ containing 4 mg/mL bovine serum albumin and 100 mIU/mL of equivalents of FSH covered with 250 μL clear mineral oil to prevent evaporation and maintain culture conditions. Total of 270 oocytes with a full or moderate attachment of cumulus cells per treatment were incubated at 38.5 °C.

### 2.6 Bull sperm preparation for *in vitro* fertilization

Frozen–thawed semen with proven fertility were purchased from American Breeders Service (Global Inc. Company). The frozen semen straws were removed from the liquid nitrogen tank (−196 °C) during thawing. The frozen semen straw was then exposed to 10 s in the air and then placed for 1 min in warm (37 °C) water. The semen straw was dried off the water with a paper towel and cut on both sealed ends and the contents inside the straw were collected into a 15 mL Falcon^®^ tube. Sperm motility was assessed using a computer-assisted sperm analysis- Sperm Class Analyzer (CASA- SCA^®^, Microptic, Spain) system at ×10 magnification. For IVF, three preparation protocols were used depending on the fertilization medium: BO-IVF^™^: Thawed semen was washed twice with 6 mL of pre-warmed BO wash medium containing caffeine, centrifuged at 1500 rpm for 8 min at 37 °C. BO IVF: Thawed semen was similarly washed twice with 6mL BO wash medium and centrifuged under the same conditions. VitroFert^™^: Semen was diluted with 4 mL of BO Semenprep^®^ medium centrifuged twice at 328 × g for 5 min at 37 °C and used for fertilization.

### 2.7 *In vitro* fertilization of cattle oocytes

Three fertilization media were evaluated: VitroFert^™^ (ART Lab Solutions, Australia; Group 1), BO-IVF (Our prepared media, Group 2) and BO-IVF^™^ (IVF Bioscience, United Kingdom; Group 3). Following IVM, oocytes were washed in five 100 μL drops of BO IVF^™^ wash medium prepared in Falcon^®^ 1008 Petri dishes and overlaid with 3 mL of clear mineral oil. Subsequently, groups of 10–15 oocytes were placed into two pre-warmed 50 μL fertilization drops of BO-IVF medium under 250 μL clear mineral oil. All media contained essential fatty acid-free bovine serum albumin and heparin to facilitate capacitation. Frozen–thawed sperm (section 2.6) was diluted to a final concentration of 1 × 10⁶ sperm/mL, and 50 μL of the suspension was added to each fertilization drop. Oocytes and sperm were co-incubated for 18 h at 39.5 °C in a humidified atmosphere of 5% CO₂. The experiment was replicated six times per treatment group.

#### 2.7.1 Experiment 1

Evaluating fertilization efficiency of vitrified cattle oocytes in different media.

Following fertilization (18 h) of oocyte-sperm incubation, portion of presumptive zygotes were removed from the IVF drops into a 1.5 mL Eppendorf tube containing 200 μL of M199 + 10% FBS medium and vortexed for 1 min to remove the cumulus cells and excess sperm while other portions were subjected to embryo culture. The pronucleus (PNs) were observed under the inverted research microscope (Olympus, IX71, Japan). The fertilization status was assessed by investigating the presence and number of PN. Typically, normal fertilized oocytes displayed the formation of two pronuclei (male and female, 2PN) within the cytoplasm. The oocyte with two PNs were considered as normal fertilization, whereas those with one and more than 2PN (indicative of polyspermy) were considered to have undergone abnormal fertilization, and those with no PN were defined as unfertilized. The total fertilization rate was determined by comparing the number of fertilized oocytes to the total number of oocytes. The normal fertilization rate was calculated by comparing the number of oocytes that form 2PN with the total number of fertilized oocytes. Meanwhile, abnormal fertilization of 1PN and more than 2PN (polyspermy) was calculated as the ratio of oocytes with less and more than 2PN to the total number of fertilized oocytes. This treatment was replicated 6 times.

#### 2.7.2 Experiment 2

To determine cleavage and blastocyst formation rates of post-warmed oocytes following fertilization in different culture media.

### 2.8 *In vitro* embryo culture in cattle presumptive zygotes

After 18 h of IVF, the cumulus cells were removed, and the embryos were transferred to different culture drops. Presumptive zygotes from BO IVF media were washed five times in 5 drops of 100 μL pre-warmed synthetic ovum fluid medium supplemented with bovine synthetic albumin (SOF-BSA) and transferred into 50 μL of SOF-BSA (BO IVC; Group 2) medium covered with clear mineral oil. The culture of presumptive zygotes from BO IVF^™^ to BO IVC^™^ (Group 3) contained 100 μL BO-IVC medium drops under clear mineral oil. The presumptive zygotes were washed 2 times in 100 μL pre-warmed BO IVC^™^ medium drops. These were then transferred into fresh 100 μL IVC drops of the same medium covered with clear mineral oil. Presumptive zygotes from VitroFert^™^ medium were washed 2 times in 380 μL of pre-warmed VitroCleave PLUS^™^ (Group 1) medium and transferred into 100 μL of VitroCleave PLUS^™^ drop covered with clear mineral oil. Presumptive zygotes from both groups of SOF BSA and BO IVC media were cultured for 48 h while presumptive zygotes from VitroCleave PLUS^™^ medium were cultured for 96 h was carried out in Nunclon^®^ cell culture dishes, placed in a modular chamber containing 5% O_2_ and 5% CO_2_ mixed gas added for a minute. On Day 2–4 of IVC, the presumptive zygotes were assessed for their developmental stages, including total cleavage, lysed embryos, 1-cell, 2–4 cell, and ≥8-cell stages, and the data were recorded accordingly. This experiment was replicated 6 times.

### 2.9 Assessment of morula and blastocyst formation in cattle oocytes in different media

During day 5 of IVC, the medium was replaced from SOF BSA to SOF-FBS (Group 2). For embryos cultured in BO IVC^™^ (Group 3), the culture medium remained unchanged throughout the incubation period. For VitroBlast (Group 1) embryos were transferred to the blastocyst culture dish after 96 h in the cleavage medium, washed in the central drop, and incubated under controlled conditions of 38.5 °C, 6% CO₂, 7% O₂, and balance N₂ for an additional 48–72 h. At the end of the IVC (Day 7–8) the embryos were evaluated for morula and blastocyst formation rates under the stereo microscope.

### 2.10 Statistical analysis

Fertilization and cleavage rates up to the blastocyst stage were analyzed using the chi-square test. Differences in the effects of post-thaw cryopreservation across fertilization and culture media were assessed by analysis of variance (ANOVA), with means compared using Tukey’s test and treatment means were further separated using Fisher’s protected t-test. Data are presented as mean ± standard deviation (SD). All data analyses were performed using SAS software (version 9.4), and statistical significance level of *p* < 0.05.

## 3 Results

### 3.1 Evaluating fertilization efficiency of post warmed cattle oocytes in different media

The fertilization outcomes of presumptive zygotes derived from vitrified and non-vitrified cattle oocytes fertilized in three different media (VitroFert^™^; Group 1, BO IVF; Group 2 and BO IVF^™^; Group 3) were assessed based on presumptive zygote developmental stages (Table 1). Among vitrified oocytes, the highest total fertilization rate was observed in presumptive zygotes fertilized with BO IVF^™^ medium (35.33 ± 3.61) followed by BO IVF (30.00 ± 3.28), which showed a statistically significant difference compared to VitroFert^™^ (28.83 ± 5.23). In non-vitrified oocytes, the highest total fertilization rate was observed in BO IVF^™^ (65.66 ± 4.96) and VitroFert^™^ (63.50 ± 3.85) compared to BO IVF (57.63 ± 3.61; *p* < 0.05). Notably, vitrification increased the proportion of lysed oocytes, particularly in VitroFert^™^ (42.16 ± 6.91) and BO IVF (39.00 ± 6.51), indicating greater post-warming damage in these media compared to BO IVF^™^ (30.00 ± 6.57). Across all three media, non-vitrified oocytes consistently exhibited higher total fertilization rates compared to their vitrified counterparts (*p* > 0.05).

**Table 1:** Comparison of presumptive zygote developmental stages (%) in vitrified and non-vitrified cattle oocytes fertilized in BO IVF, Vitrofert^™^ and B0 IVF^™^ medium.

Treatment	Type of oocytes	None	Lysed	1 PN	2PN	≥2PN	Total FR
BO IVF	Non-vitrified	30.00 ± 3.28	12.16 ± 4.83^c^	15.33 ± 3.61^a^	30.16 ± 5.38^a^	12.16 ± 4.83^bc^	57.66 ± 3.61^b^
	Vitrified	31.16 ± 9.13	39.00 ± 6.51^a^	10.00 ± 3.28^b^	12.16 ± 4.83^bc^	7.66 ± 6.37^cd^	30.00 ± 3.28^d^
Vitrofert^™^	Non-vitrified	31.16 ± 7. 98	11.16 ± 13.25^c^	15.33 ± 3.61^a^	30.00 ± 3.28^a^	18.83 ± 5.26^ab^	63.50 ± 3.85^a^
	Vitrified	28.83 ± 7.98	42.16 ± 6.91^a^	13.33 ± 5.81^ab^	9.00 ± 3.09^c^	6.50 ± 7.12^cd^	28.83 ± 5.23^d^
BO IVF^™^	Non-vitrified	28.83 ± 5.23	5.66 ± 4.96^c^	17.66 ± 3.61^a^	27.83 ± 4.83^a^	20.16 ± 7.30^a^	65.66 ± 4.96^a^
	Vitrified	34.33 ± 7.81	30.00 ± 6.57^b^	16.50 ± 3.83^a^	14.40 ± 3.13^b^	3.50 ± 3.83^d^	35.33 ± 3.61^C^

^a-d^Different superscripts within a stage indicate significant differences (*p* < 0.05). PN = pronucleus, FR = fertilization rate.

**Figure 1:**
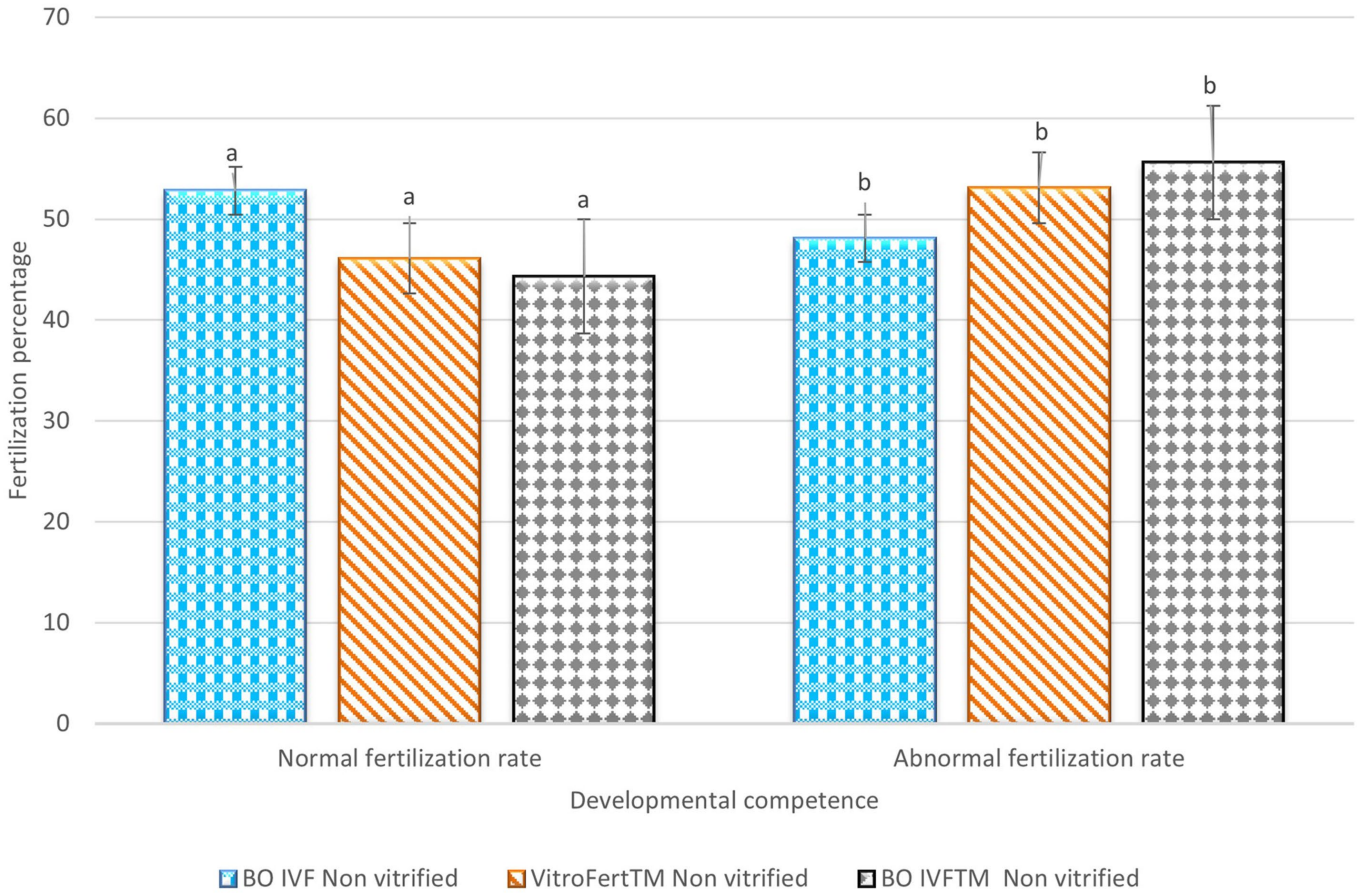
Fertilization outcomes of presumptive zygote derived from non-vitrified cattle oocytes in different media. The percentages of normal and abnormal fertilization rates are presented for oocytes vitrified and fertilized in BO IVF, VitroFert^™^, and BO IVFTM media. Data was expressed as mean ± SEM. Different superscripts (a–b) within each fertilization category indicate significant differences (p < 0.05).

The fertilization outcomes of presumptive zygotes derived from non-vitrified presumptive zygotes fertilization media is shown in Figure 1. The BO-IVF reported the highest proportion of normal fertilization (2PN; 52.83 ± 7.90) which was significantly higher than that observed with VitroFert^™^ (46.16 ± 5.60) and BO-IVF^™^ (44.33 ± 12.53) media (*p* < 0.05). Contrarily, BO-IVF^™^ (55.66 ± 12.53) and VitroFert^™^ (53.16 ± 6.33) exhibited higher abnormal fertilization rates (1PN+ > 2PN; Figure 1) compared to BO-IVF (48.16 ± 8.68; *p* < 0.05).

**Figure 2:**
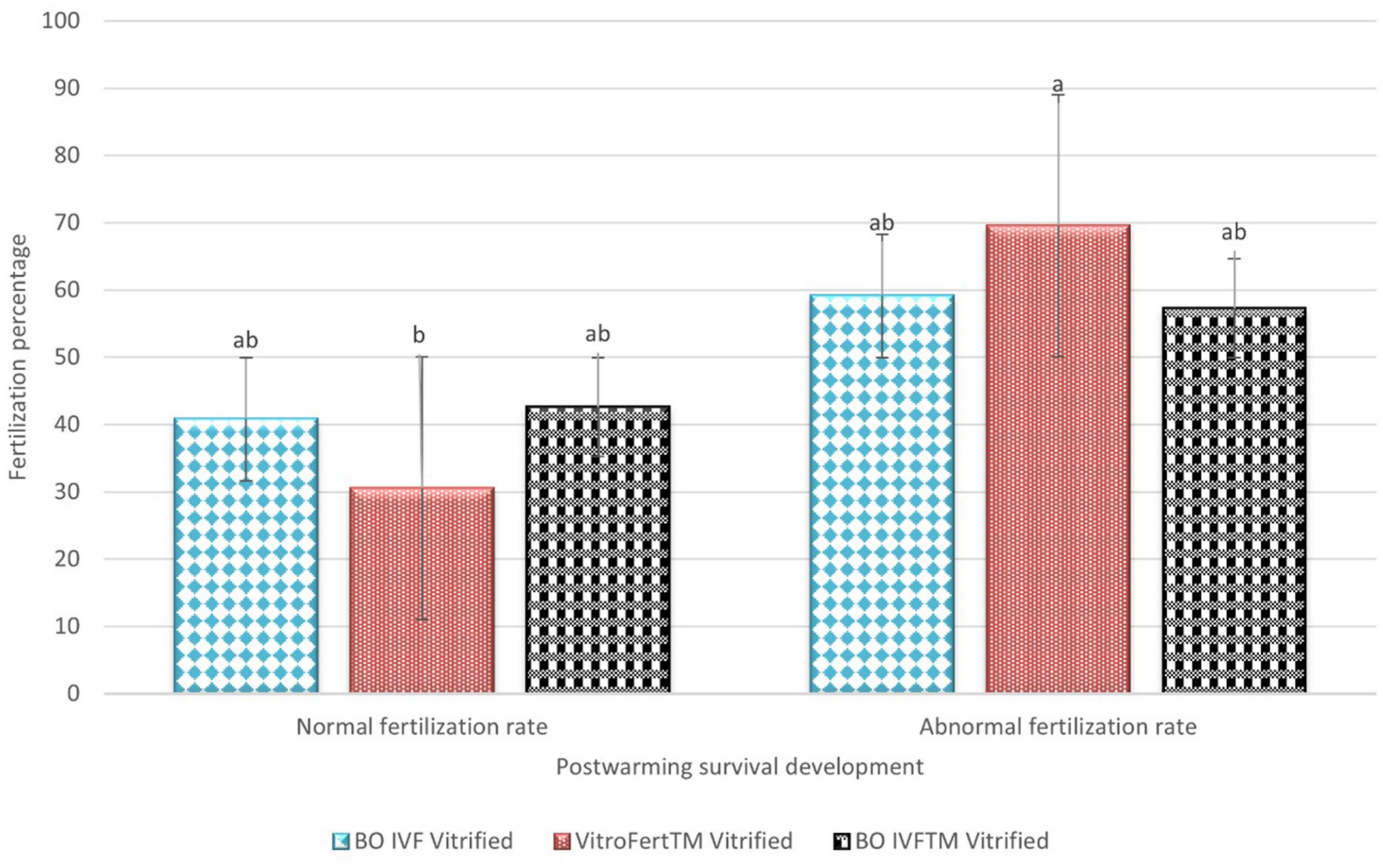
Post-warming fertilization outcomes of vitrified cattle oocytes in different media. The percentages of normal and abnormal fertilization rates are presented for oocytes vitrified and fertilized in BO IVF, VitroFert^™^, and BO IVFTM media. Data was expressed as mean ± SEM. Different superscripts (a–b) within each fertilization category indicate significant differences (*p* < 0.05).

**Figure 3:**
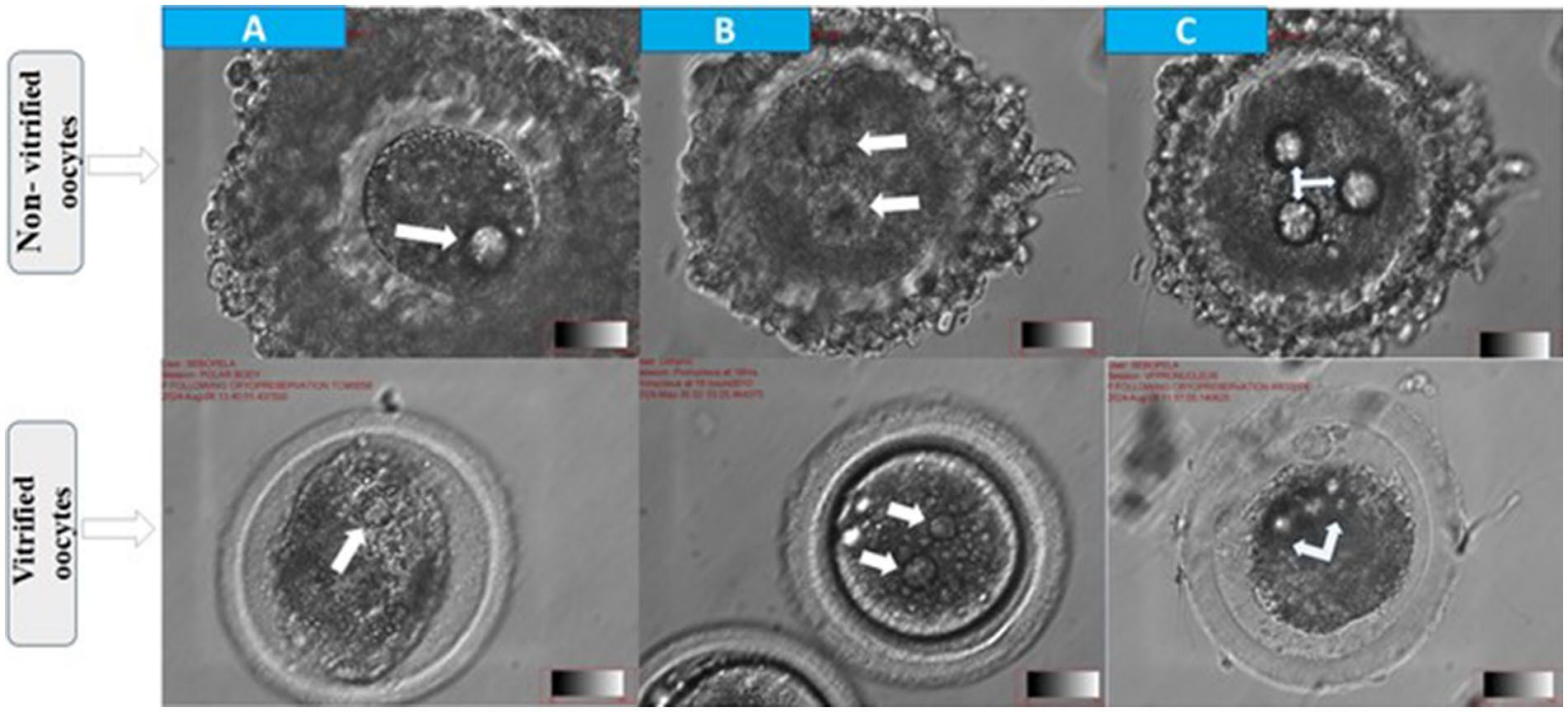
Morphological assessment of fertilization status in cattle oocytes after 18 h of in vitro fertilization. Top panel: non-vitrified oocytes; bottom panel: vitrified oocytes **(A)** abnormal fertilization with one visible pronucleus (arrow) **(B)** normal fertilization indicated by the presence of two pronuclei (arrows) and **(C)** polyspermy characterized by ≥2 pronuclei (arrows).

The fertilization outcomes of presumptive zygote derived from vitrified oocytes is indicated in Figure 2. The highest normal fertilization rate (2PN) among vitrified oocytes was observed in presumptive zygotes fertilized with BO IVF^™^ medium (42.66 ± 9.43) and BO-IVF (40.83 ± 15.62), both of which were significantly different compared to VitroFert^™^ (30.50 ± 8.45). Abnormal fertilization was generally higher across all media, with VitroFert^™^ showing the highest (69.50 ± 8.45), followed by BO IVF (59.16 ± 15.62) and BO IVF^™^ (57.33 ± 9.43; Figure 3). VitroFert^™^ differed significantly from the others, indicating a higher tendency for abnormal fertilization events in this medium. Overall, while BO IVF^™^ supported relatively better normal fertilization after vitrification, all media exhibited a higher rate of abnormal fertilization compared to normal fertilization.

### 3.2 To determine cleavage and blastocyst formation rates of post-warmed oocytes following fertilization in different culture media

**Figure 4:**
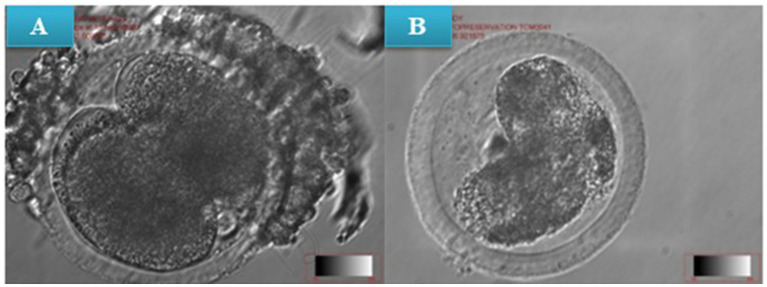
**(A)** In vitro cleavage of non-vitrified (pre-warmed) presumptive zygotes, **(B)** In vitro cleavage of vitrified presumptive zygote (post-warmed) at x10 magnification.

The evaluation of embryonic development in non-vitrified and vitrified oocytes subjected to different culture media treatment (VitroCleave PLUS^™^; Group 1, BO IVC; Group 2, BO IVC^™^; Group 3) is presented in Table 2. Across all three media, non-vitrified oocytes consistently achieved higher total cleavage rates (38.83–55.28%; Figure 4) compared to vitrified counterparts (26.50–30.00%, *p* > 0.05). Non-vitrified oocytes showed greater progression to advanced cleavage stages (≥8-cell) and reduced lysis rates, reflecting enhanced developmental competence. In BO IVC medium, non-vitrified oocytes yielded 22.33% at the 2–4-cell stage and 17.66% at the ≥8-cell stage, whereas vitrified oocytes showed a markedly higher incidence of lysis (40.00 ± 6.26; Group 3) and reduced ≥8-cell embryos (8.83 ± 5.23). VitroCleave PLUS^™^ recorded the highest 2–4-cell development in non-vitrified oocytes (35.50 ± 6.97; Group 1), with a total cleavage rate of 54.33%. However, vitrified oocytes in this medium displayed elevated lysis (45.50 ± 6.41) and a reduced ≥8-cell proportion (13.16 ± 4.11; *p* > 0.05) percentages. In BO IVC^™^ medium, non-vitrified oocytes achieved the highest overall cleavage (55.28 ± 7.40; Group 3) and the largest proportion of ≥8-cell embryos (24.71 ± 7.40) percentages.

**Table 2:** Comparison of embryonic development stages between non-vitrified and vitrified cattle oocytes cultured in three different media.

Treatment	Type of the oocyte	1 cell	Lysed	2–4 cell	≥8 cell	Total cleavage rate
BO IVC	Non-vitrified	41.16 ± 5.26^a^	18.83 ± 5.26^c^	22.33 ± 3.61^b^	17.66 ± 3.61^b^	38.83 ± 5.26^b^
	Vitrified	37.83 ± 8.20^a^	40.00 ± 6.26^b^	13.00 ± 0.00^c^	8.83 ± 5.23^c^	27.83 ± 4.83^c^
VitroCleave PLUS^™^	Non-vitrified	37.50 ± 8.09^a^	7.67 ± 6.37^d^	35.50 ± 6.97^a^	18.83 ± 5.26^ab^	54.33 ± 6.65^a^
	Vitrified	24.50 ± 6.97^b^	45.50 ± 6.41^ab^	15.50 ± 5.39^c^	13.16 ± 4.11^bc^	30.00 ± 3.28^c^
BO IVC^™^	Non-vitrified	38.00 ± 8.24^a^	5.28 ± 4.64^d^	30.57 ± 7.45^a^	24.71 ± 7.40^a^	55.28 ± 7.40^a^
	Vitrified	26.83 ± 4.11^b^	48.83 ± 7.98^a^	14.33 ± 4.96^c^	10.16 ± 5.38^C^	26.50 ± 7.12^c^

^a-d^Different superscripts within a stage indicate significant differences (*p* < 0.05).

By contrast, vitrified oocytes exhibited the highest presumptive zygote lysis rate in the study in BO IVC (40.00 ± 6.26), VitroCleave PLUS^™^ (45.50 ± 6.41) and BO IVC^™^ (48.83 ± 7.98). Consequently, these groups demonstrated the lowest cleavage progression percentages (*p* < 0.05). Overall, vitrification had a negative effect on cleavage progression and embryo survival, and the impact differed depending on the culture medium. VitroCleave PLUS^™^ supported relatively better post-vitrification development than BO IVC^™^ and BO IVC, but none fully mitigated the detrimental impact of cryopreservation on oocyte viability and developmental competence.

**Figure 5:**
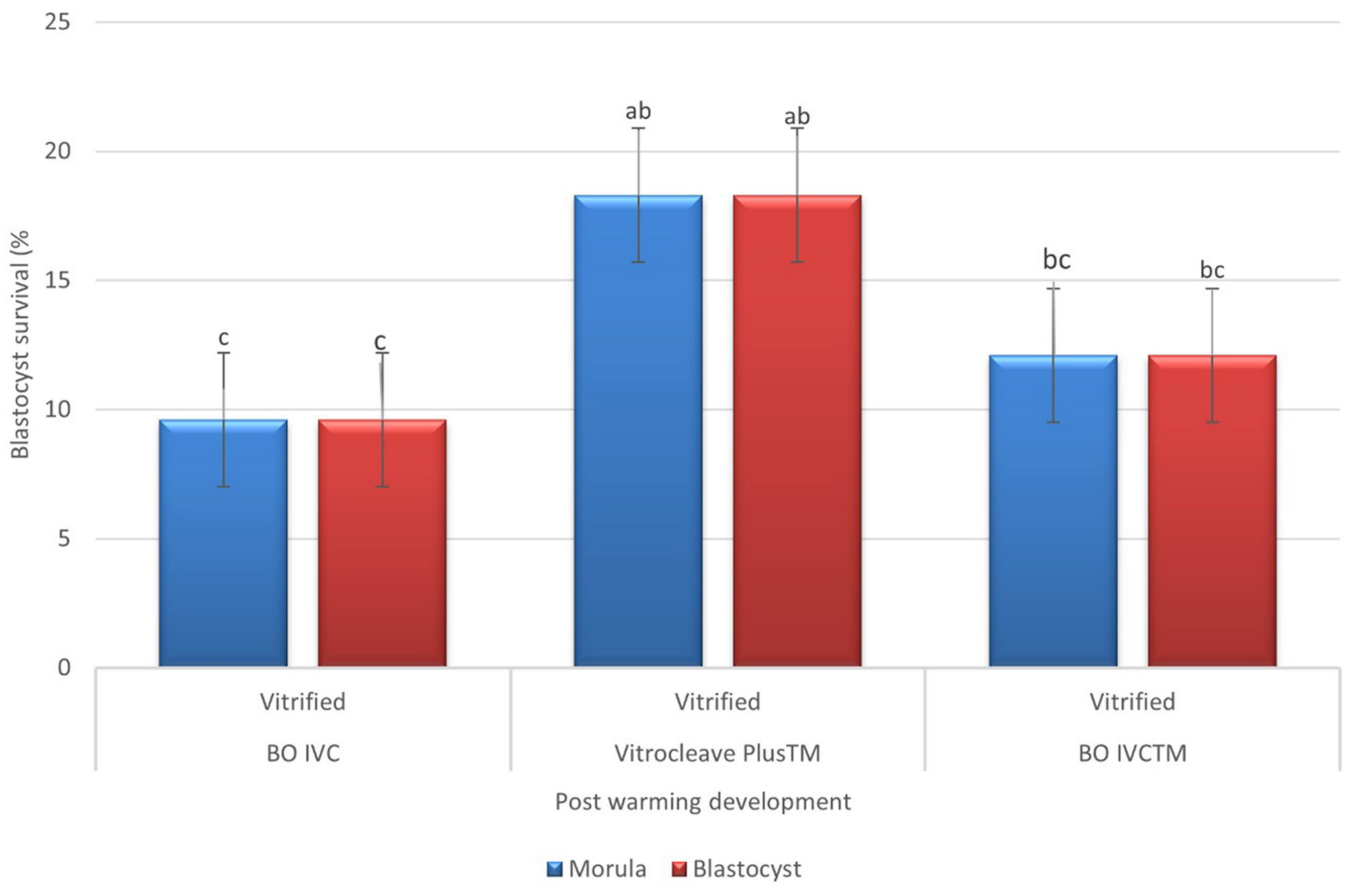
Post-warming development of vitrified bovine oocytes in different culture media. The percentages of morula (blue bars) and blastocyst (red bars) survival following vitrification and warming are shown for BO IVC, VitroCleave Plus^™^, and BO IVCTM media. Values are presented as mean ± SEM. Different superscripts (a–c) shows significant differences (*p* < 0.05) among groups within the same developmental stage.

Post-warming developmental competence of presumptive zygote cultured in different media is shown in Figure 5. Embryos cultured in Vitrocleave Plus^™^ demonstrated the highest developmental rates, with morula (18.33 ± 15.7) and blastocyst formation (18.33 ± 15.7) reaching, respectively. These rates were significantly greater than those observed in embryos cultured in BO IVC^™^, which showed morula development and blastocyst development as 12.16%. Embryos cultured in BO IVC exhibited the lowest developmental rates for both morula (9.66 ± 15.18) and blastocyst (9.66 ± 15.18) stages. Overall, VitroCleave Plus^™^ yielded intermediate developmental outcomes between BO IVC^™^ and BO IVC (*p* < 0.05).

**Figure 6:**
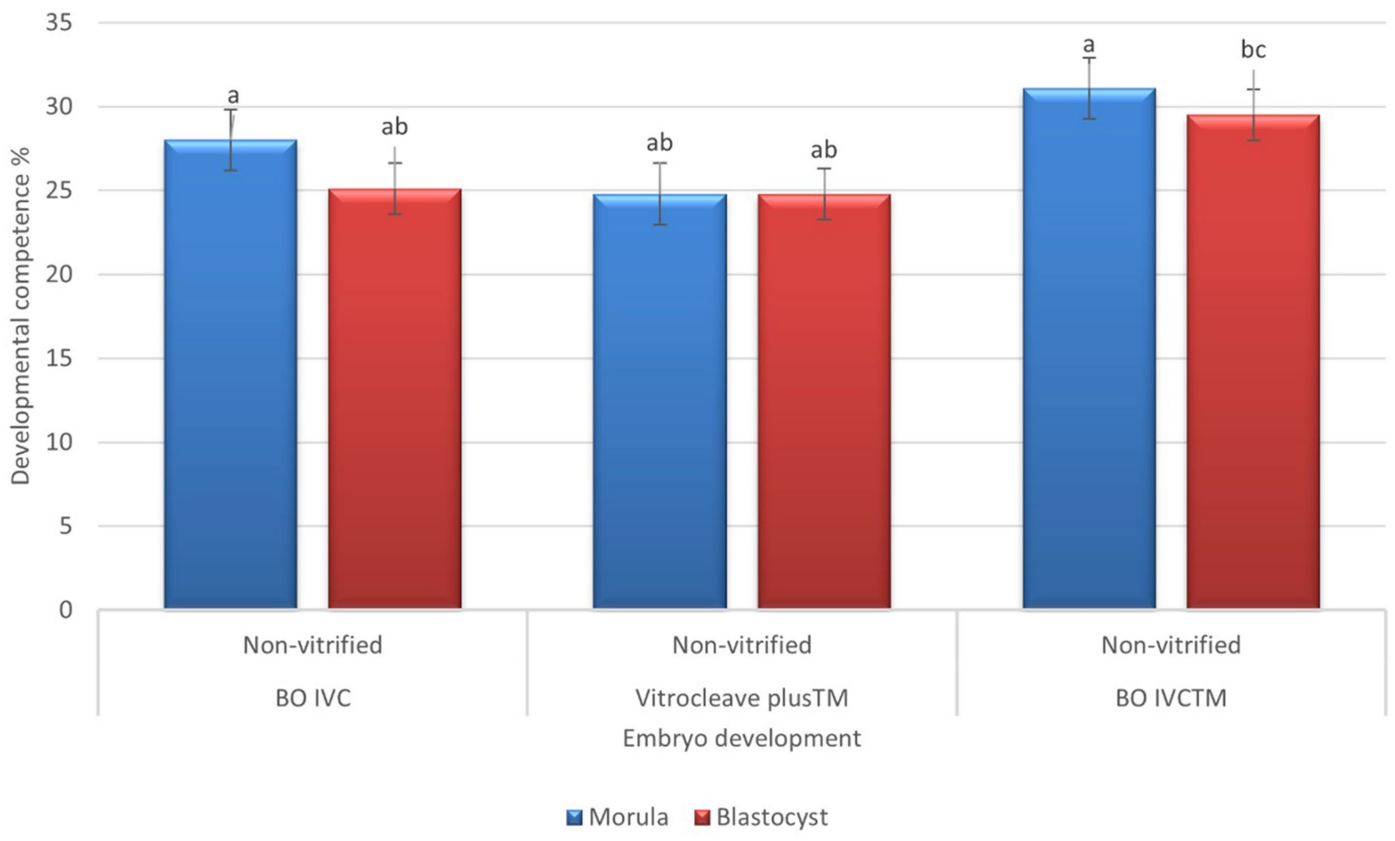
Developmental competence of presumptive zygotes derived from non-vitrified cattle oocytes cultured in different media. The percentages of morula (blue bars) and blastocyst (red bars) survival are shown for BO IVC, VitroCleave Plus^™^, and BO IVCTM media. Values are presented as mean ± SEM. Different superscripts (a–c) shows significant differences (*p* < 0.05) among groups within the same developmental stage.

The developmental competence of presumptive zygote cultured derived from non-vitrified oocytes in different media is shown in Figure 6. There was no statistically significant differences in embryo development across the three-culture media. However, embryos cultured in BO IVC^™^ (31.14 ± 6.17) achieved the highest morula-stage development, followed by BO IVC (28.00 ± 6.38) and VitroCleave Plus (24.83 ± 8.70; *p* > 0.05). A similar pattern was observed at the blastocyst stage, where BO IVC^™^ again yielded the highest developmental rate (29.57 ± 6.97) with BO IVC (25.16 ± 7.41) and VitroCleave Plus^™^ (24.83 ± 8.70; *p* > 0.05) showing lower but comparable outcomes. Although the differences were not significant, these results suggest that BO IVC^™^ may provide a slightly more supportive environment for both morula and blastocyst development compared to the other tested media.

## 4 Discussion

The successful recovery of viable embryos from cryopreserved oocytes continues to be a major challenge across most mammalian species (22–24). Although several commercial vitrification protocols exist differing in cryoprotectant types and concentrations and exposure conditions, their effectiveness differs considerably. In this study, we present the first evaluation in South Africa condition of the newly introduced ART Lab Solutions kit for bovine oocyte cryopreservation through to embryo development. Assessing this medium is important because the choice of vitrification protocol and cryoprotectant composition directly influences oocyte survival, fertilization success, and subsequent embryonic development. The results demonstrated that vitrification negatively influenced fertilization, cleavage, and blastocyst potential, regardless of the medium used. Similarly, Angel-Vélez et al. (12) found that vitrification of bovine oocytes impaired embryo development (lower proportion reaching later cleavage and blastocyst stages), although lower cryoprotectant concentration in equilibration solutions seems to be less detrimental for embryo yield. Non-vitrified oocytes consistently showed higher fertilization and cleavage rates, as well as a greater proportion of advanced-stage embryos (≥8-cell), compared to vitrified oocytes. These findings highlight the persistent detrimental effects of cryopreservation on cellular integrity, meiotic spindle organization, zona pellucida biomechanics, and cytoplasmic maturation. Commonly, sucrose has been accepted as a highly effective non-permeating saccharide in removing intracellular CPAs (DMSO, EG, GLY) and enabling stepwise rehydration to support isotonic equilibration (10). The study by Jin and Mazur (25) demonstrated in mouse oocytes that osmotic dehydration before cooling and an appropriate warming rate are critical for cryopreservation success. Similarly, our findings further support this principle by showing that optimized warming media and conditions are essential to preserve fertilization and developmental competence. Additionally, our findings suggest that cattle oocytes are sensitive to variations in sucrose concentrations and temperature during the warming process. The observed tolerance to these conditions reflects their inherent adaptability; however, when this adaptability is exceeded, fertilization, cleavage, and blastocyst development are compromised. A key factor contributing to reduced fertilization is the structural and functional compromise of cumulus–oocyte complexes. The success of immature oocyte cryopreservation largely depends on maintaining the integrity of both the oocyte and its surrounding cumulus cells (8, 26). Gap junctions between cumulus cells and the oocyte provide essential metabolic support during maturation and fertilization (27). However, cryopreservation reduces the number of cumulus cell layers, which may explain the lower fertilization rates and reduced blastocyst yield observed in this study. Since cumulus cells also facilitate sperm trapping, selection, and prevention of premature zona hardening (28, 29), their loss compromises fertilization efficiency. In line with this, we observed that presumptive zygotes fertilized with BO IVF^™^ and VitroFert^™^ exhibited higher incidences of polyspermy compared to BO IVF^™^ alone following vitrification. Cryopreserved oocytes often exhibit altered cortical granule distribution and premature zona hardening, leading to abnormal fertilization outcomes such as polyspermy (30). Interestingly, BO IVF^™^ supported higher normal fertilization rates post-cryopreservation compared to the other treatments, suggesting that this medium may better mitigate some of the deleterious effects of vitrification. When considering embryo development, non-vitrified oocytes cultured in BO IVC^™^ (IVF Biosciences) and VitroCleave PLUS^™^ (ART Lab solutions) achieved higher cleavage and advanced developmental stages than vitrified oocytes. Similarly, Nielsen et al. (31) concluded that the developmental rates and gene expression of *in vitro* produced bovine blastocysts were affected by the use of different culture media increased blastocyst rates, apparently superior embryo quality, and more abundant gene expression were achieved when blastocysts were cultured in BO-IVC culture media (IVF Biosciences) compared with SOF.

The higher proportion of lysed oocytes in vitrified groups suggests that cryoinjury impairs plasma membrane integrity, spindle function, and mitochondrial activity, ultimately reducing embryonic genome activation. Notably, BO IVC^™^ and VitroCleave PLUS^™^ appeared more supportive of early embryonic development in non-vitrified oocytes than BO IVC, but these advantages were lost following vitrification. Similarly, a study by Hajian et al. (32) reported that the blastocyst rate in the BO medium was higher than that observed in the SOF medium. Thus, medium optimization alone cannot fully overcome cryoinjury. Our findings contrast with earlier studies showing improved blastocyst yields in SOF media supplemented with BSA or FCS (33). Collectively, the results suggest that although BO IVF^™^ provides the most supportive environment for both vitrified and non-vitrified oocytes minimizing oocyte lysis and maintaining higher fertilization rates, vitrification significantly compromises developmental potential. In this study, blastocyst development from vitrified oocytes was low in all treatments, showing that vitrification still reduces developmental competence even with improvements in fertilization and culture methods. This study contradicts with results found by Yagoub et al. (34), who found higher cleavage and blastocyst survival in vitrified oocytes using Pods and the Garage system and cultured with VitroCleave PLUS^™^. The difference may be due to the methods used, as our study relied on a conventional straw-based protocol, while Yagoub et al. (34) used a nanoliter device designed to improve cooling and warming efficiency. Improving outcomes will require an integrated approach: optimizing cryoprotectant selection, cooling/warming rates, fertilization medium, and culture conditions. Our observations, combined with recent findings on simplified warming procedures, demonstrate that warming of oocytes is possible under different conditions and opens possibilities for further optimization of laboratory procedures. The use of different warming solutions and exposure times results in different clinical outcomes with effect on developmental competence on cattle oocytes following fertilization. These findings are particularly relevant in the context of germplasm and tissue cryobanks, which aim not only to conserve genetic resources but also to enhance reproductive efficiency in livestock (35, 36). However, outcomes of embryo development following oocyte vitrification remain highly variable among studies (24, 37). This variability arises from differences in vitrification protocols, IVM and IVF systems, culture media, laboratory environments, local climatic conditions, donor breeds, and biological material quality (38).

## 5 Conclusion

Among the protocol tested, the BO IVF^™^ medium (IVF Biosciences) demonstrated potential for enhancing post-warming oocyte competence and fertilization outcomes compared to other treatment groups. Overall, these findings indicate that vitrification significantly compromises fertilization, cleavage, and developmental rates, and that VitroCleave Plus^™^ (ART Lab Solutions) offers the most favourable post-warming culture environment for supporting cattle embryo development to the blastocyst stage.

## Data Availability

The original contributions presented in the study are included in the article/supplementary material, further inquiries can be directed to the corresponding author.
